# Neutrophils fertilize the pre-metastatic niche

**DOI:** 10.18632/aging.102258

**Published:** 2019-09-10

**Authors:** WonJae Lee, Honami Naora

**Affiliations:** 1 Department of Molecular and Cellular Oncology, University of Texas MD Anderson Cancer Center, Houston, TX 77030, USA

**Keywords:** metastasis, niche, neutrophils, extracellular traps

The concept of the pre-metastatic niche originates from Paget’s ‘seed and soil’ hypothesis which postulated that cancer cells are predisposed to metastasize to specific sites that are conducive for colonization. It is increasingly recognized that permissive microenvironments at destination sites are cultivated prior to cancer cell implantation through cooperativity between tumors and a variety of host cells. Neutrophils are the most abundant type of leukocyte and act as ‘first responders’ to pathological insults. Although there is compelling evidence that neutrophils are recruited to pre-metastatic niches such as the lung [1,2], the role of neutrophils in niche establishment has been unclear. For example, it has been reported that neutrophils in the pre-metastatic lung niche are cytotoxic to cancer cells [1], but can also initiate metastasis at this site by producing inflammatory mediators [2].

In a recent study [3], we investigated the cellular dynamics in the omentum, a fat pad that suspends from the stomach and is the predominant site of metastasis of ovarian cancer. A striking feature of the omentum, that distinguishes this tissue from other fat pads, is its abundance of highly vascularized immune cell structures called milky spots that are predominantly composed of lymphocytes and macrophages. We found that ovarian tumors, generated either orthotopically or via i.p. injection, colonized the omenta of nude mice and NOD-*scid* IL2Rγ^null^ mice as effectively as in immunecompetent mice [3]. This observation implicated non-lymphoid immune cell constituents in directing tropism of ovarian cancer cells for the omentum. Whereas the abundance of macrophages in the omenta of mice did not significantly change during the pre-metastatic stage, neutrophils increased 6-to-7 fold and were localized to specialized vessels called high endothelial venules in milky spots [3]. Depletion of neutrophils from mice did not significantly affect growth of primary tumors but reduced omental metastasis by 70% [3]. These findings indicated that neutrophil influx into the omentum is an early prerequisite step for the formation of ovarian tumor implants at this site.

Further characterization of neutrophils in our study revealed that neutrophils respond to ovarian tumors by undergoing a unique form of cell death in which chromatin fibers called neutrophil extracellular traps (NETs) are extruded [3]. Several ovarian tumor-derived chemokines were identified to potentiate NET formation including interleukin-8, growth-regulated oncogene (GRO)-α, GRO-β and granulocyte-colony stimulating factor [3]. NET formation was discovered in 2004 as a mechanism by which neutrophils capture and disarm microbes [4]. More recently, NETs have been detected in patients with advanced-stage metastatic breast cancer [5], and thought to promote metastasis by trapping circulating cancer cells or by awakening dormant cancer cells through remodeling the extracellular matrix [6,7]. The implication that emanates from these studies is that NETs act as an effector mechanism at a relatively late stage in the metastatic cascade. Notably in our study, NETs were detected in the omenta of women with stage I or stage II ovarian cancer (i.e. disease that is ovarian-confined or locally extended to adjacent pelvic structures) and in the omenta of ovarian tumor-bearing mice prior to colonization of this site [3]. Our findings implicate NET formation in the pre-metastatic omental niche as an early host response to the presence of primary ovarian tumors.

To investigate the functional significance of NETs in omental metastasis, we generated mice with neutrophil-specific deficiency of peptidylarginine deiminase 4 (PAD4), an enzyme that is essential for NET formation, and evaluated the progression of orthotopic ovarian tumors in these mice (i.e. *Padi4*^-/-^). *Padi4*^-/-^ mice were defective in NET formation but had normal leukocyte counts and normal neutrophil chemotactic responses [3]. Strikingly, omental metastasis but not primary tumor growth was reduced by 70% in *Padi4*^-/-^ mice [3]. Furthermore, omental colonization was reduced by 70% when NET-competent mice were treated with a PAD4 small molecule inhibitor that inhibited NET formation or with recombinant DNase that degraded NETs [3]. Collectively, our findings support a model in which omental metastasis of ovarian cancer is orchestrated by the induction of NET formation in the pre-metastatic omental niche that renders this site fertile for implantation of circulating cancer cells.

Our findings regarding the significance of NETs in forming the pre-metastatic niche and in directing metastatic tropism may have important implications for cancer treatment. Surgical removal of the omentum is a standard procedure for staging and treatment of ovarian cancer irrespective of whether the omentum is involved. However, the omentum plays an important role in peritoneal defense and clinical studies of women with stage I/II ovarian cancer have questioned the benefit of omentectomy in the absence of overt metastasis [8]. Although NET-inhibiting agents may have limited therapeutic utility in cases where metastasis is overt, these agents might be beneficial for preventing metastasis to uninvolved sites that are not resectable or whose functions need to be preserved.
